# A predictive model for clinical pregnancy following single Day-6 blastocyst transfer in frozen-thawed embryo transfer cycles

**DOI:** 10.3389/fendo.2024.1428086

**Published:** 2024-11-22

**Authors:** Lidan Liu, Qiuying Gan, Yihua Yang, Bo Liu, Qianyi Huang, Mujun Li

**Affiliations:** ^1^ Guangxi Reproductive Medical Center, The First Affiliated Hospital of Guangxi Medical University, Nanning, China; ^2^ Reproductive Center, Nanning Maternity and Child Health Hospital, Nanning, China

**Keywords:** frozen-thawed cycles, single Day 6 blastocyst transfers, predictive model, clinical pregnancy, nomogram

## Abstract

**Purpose:**

This study aimed to develop a predictive model for assessing clinical pregnancy probabilities in patients undergoing frozen-thawed cycles with single Day 6 blastocyst transfers.

**Methods:**

We conducted a two-center retrospective cohort study analyzing 1,381 frozen-thawed single Day 6 blastocyst transfer cycles from June 2016 to December 2022. The primary outcome was the clinical pregnancy rate per cycle. Data were divided into training, testing, and validation groups in a 6:2:2 ratio. Univariate and LASSO regression analyses identified factors influencing clinical pregnancy, which were incorporated into a multiple regression model to predict outcomes. Model performance was assessed in terms of discrimination, calibration, and clinical utility.

**Results:**

Factors independently predicting clinical pregnancy included inner cell mass (ICM) grade, trophectoderm (TE) grade, Day 3 (D3) fragmentation, endometrium thickness, and male age at oocyte pick-up (OPU). The AUC values for the training, testing, and validation sets were 0.66, 0.65, and 0.60, respectively, indicating acceptable performance. Calibration curves demonstrated good predictive accuracy, with slopes of 0.988, 0.871, and 1.263 for the respective groups.

**Conclusion:**

The developed nomogram accurately predicts clinical pregnancy probabilities in patients undergoing single Day 6 blastocyst transfers in frozen-thawed cycles, enhancing clinical decision-making by integrating crucial embryological and clinical parameters.

## 1 Introduction

Embryo transfer at the blastocyst stage has become increasingly popular and is widely adopted in assisted reproductive technology (ART) centers worldwide (1, 2). This strategy offers several clinical advantages, including improved implantation rates and reduced time to pregnancy by facilitating better embryo selection (3, 4). Compared to cleavage-stage embryos, blastocysts undergo more rigorous natural selection during culture, weeding out embryos with limited developmental potential (3, 4). As blastocyst transfers promote single-embryo transfer (SET), they reduce the risks associated with multiple pregnancies—such as gestational hypertension, preterm birth, and low birth weight—thereby improving patient safety and lowering healthcare costs (5). Advances in culture media and laboratory protocols have further facilitated the widespread adoption of both fresh and frozen-thawed blastocyst transfers, improving clinical outcomes.

While Day 5 (D5) blastocysts are considered ideal for transfer, many embryos reach the blastocyst stage on Day 6 (D6) or later under the same culture conditions (6). Despite being blastocysts, these embryos exhibit slower developmental kinetics, which are linked to varying clinical outcomes (7).Systematic reviews have shown that D5 blastocysts achieve higher clinical pregnancy and live birth rates than D6 blastocysts, both in fresh and frozen cycles (8). These differences are attributed to two key factors: (1) intrinsic disadvantages in implantation potential for slower-developing embryos, and (2) suboptimal synchronization between the endometrium and blastocyst, particularly in fresh Day 6 transfers (7, 8). Nevertheless, frozen-thawed Day 6 blastocysts (D6-FET) play a critical role in ART, especially for patients whose embryos do not reach the blastocyst stage until Day 6 (9).Recent evidence suggests that with appropriate selection and preparation, D6-FET can result in clinical outcomes comparable to D5-FET (10, 11).Thus, these embryos remain a crucial option in individualized reproductive care.

In ART, clinical outcomes such as implantation and pregnancy rates are influenced by multiple variables, including blastocyst quality, maternal age, body mass index (BMI), endometrial thickness, and hormonal preparation protocols (12–15). These factors often interact in complex ways, making it essential to apply advanced statistical methods to accurately assess their combined effects. Multivariable logistic regression is one of the most widely used tools in ART research, as it allows researchers to examine the influence of multiple independent variables on a binary outcome, such as whether a clinical pregnancy occurs (yes/no).

Multivariable logistic regression is particularly valuable because it can control for confounding variables while identifying independent predictors of clinical outcomes. For example, studies have demonstrated that variables such as maternal age, blastocyst quality, and endometrial thickness each have a significant impact on the likelihood of achieving a pregnancy, even when accounting for other factors (16). Additionally, regression models provide odds ratios (ORs), which offer a quantitative measure of the strength of association between predictors and outcomes. One limitation of logistic regression is its reliance on linear relationships between the predictors and the log odds of the outcome (17). However, when carefully specified and validated, logistic regression models remain a robust tool for ART studies. Modern studies often combine stepwise selection methods or use regularization techniques (e.g., LASSO) to identify the most relevant predictors, reducing the risk of overfitting (9).

Our study aims to develop a predictive model to estimate the probability of clinical pregnancy following single Day 6 blastocyst transfers in frozen-thawed embryo transfer (FET) cycles. Using multivariable logistic regression, we will incorporate key patient characteristics, clinical indicators, and laboratory markers to assess their combined effects on pregnancy outcomes. The model will help provide precise, evidence-based treatment recommendations, supporting clinicians in personalized decision-making and enhancing the success rates of ART.

## 2 Materials and methods

### 2.1 Study design and patients

This retrospective cohort study was conducted at two reproductive medicine centers: the First Affiliated Hospital of Guangxi Medical University and the Nanning Maternity and Child Health Hospital. It involved an analysis of 1381 consecutive frozen-thawed single Day 6 blastocyst transfer cycles between June 2016 and December 2022.Details of the ART treatments were documented in the ART database according to the Technical Standard for Human-Assisted Reproduction prescribed by the Chinese Ministry of Health. The study included patients who underwent a frozen single D6 blastocyst transfer during this period. Preimplantation genetic testing was not performed at these centers; therefore, none of the patients in this study underwent such testing. The study’s protocol received approval from the Institutional Review Board of the respective hospitals.

### 2.2 Ovarian stimulation and oocyte insemination

There were no specific restrictions on ovarian stimulation protocols. The initial dose of recombinant follicle-stimulating hormone (rFSH) was tailored based on the woman’s age, BMI, baseline FSH levels, and antral follicle counts (18). Administration of human chorionic gonadotropin (hCG) occurred when at least one follicle reached 18 mm or larger. Oocyte retrieval was carried out via vaginal ultrasound 36 hours after hCG administration. Using either IVF or ICSI based on semen quality on the day of retrieval, following our center’s routine protocol.

### 2.3 Embryo culture and blastocyst scoring

The blastocysts were cultured continuously in a single culture medium throughout all developmental stages and incubated under oil at 37°C in an environment containing 5% O_2_ and 6% CO_2_, with nitrogen as the balance gas. Blastocyst assessments were conducted using the Gardner scoring system (19).

### 2.4 Blastocyst vitrification and thawing procedures

Fully expanded blastocysts were artificially shrunk using laser before being cryopreserved using vitrification kits (KITAZATO). The embryos were then loaded onto a cryotop on day 6 post-insemination. The cryopreserved blastocysts were stored in liquid nitrogen until they were ready to be warmed. Blastocyst warming was conducted using warming kits (KITAZATO) once the endometrium achieved adequate thickness. The survival of the blastocyst was assessed by its re-expansion two hours post-warming.

### 2.5 Endometrial preparation and blastocyst transfer

Endometrial preparation for frozen embryo transfer (FET) was conducted using various protocols, including modified natural cycles (mNC), mild stimulation (MS), and hormone replacement therapy (HRT) with or without GnRH agonist pretreatment. A warmed blastocyst was transferred into the uterus under the guidance of abdominal ultrasound (20).

### 2.6 Clinical outcomes

The primary outcome was clinical pregnancy, defined as the presence of a gestational sac with a detectable heartbeat confirmed by ultrasound four weeks after a single D6 blastocyst transfer.

### 2.7 Statistical analysis

We performed statistical analysis using R software (Version 4.2.2). Participant characteristics were summarized using means and standard deviations for continuous variables, and frequencies and percentages for categorical variables. We employed t-tests to compare differences between continuous variables, and chi-square tests or Fisher’s exact tests for categorical variables. Univariable logistic regression analysis and LASSO regression were utilized to screen variables associated with clinical pregnancy. Subsequently, we conducted multivariable logistic regression to identify significant prognostic factors related to clinical pregnancy. Additionally, a nomogram was created to visually represent these prognostic factors and assist users in calculating probabilities. Model performance was evaluated based on three dimensions: discrimination, calibration, and clinical utility. Discrimination was assessed using the area under the curve (AUC) of the receiver operating characteristic (ROC) curve. Calibration was evaluated using calibration curves and unreliability tests. The clinical utility of the nomogram was assessed using decision curve analysis (DCA) by quantifying the standardized net benefit at different threshold probabilities. All reported statistical significance levels were two-sided, with a significance level set at 0.05.

Univariable logistic regression analysis and LASSO regression were utilized to screen variables associated with clinical pregnancy. Subsequently, we conducted multivariable logistic regression to identify significant prognostic factors related to clinical pregnancy. Additionally, a nomogram was created to visually represent these prognostic factors and assist users in calculating probabilities. Model performance was evaluated based on three dimensions: discrimination, calibration, and clinical utility. Discrimination was assessed using the area under the curve (AUC) of the receiver operating characteristic (ROC) curve. Calibration was evaluated using calibration curves and unreliability tests. The clinical utility of the nomogram was assessed using decision curve analysis (DCA) by quantifying the standardized net benefit at different threshold probabilities.

## 3 Results

### 3.1 Baseline characteristics

A total of 1,381 single Day 6 blastocyst transfer cycles were included in this study. The cycles were randomly divided into three groups: training group (N = 828), testing group (N = 277), and validation group (N = 276), following a 6:2:2 ratio for model development, testing, and validation. The baseline characteristics of the study population are summarized in **Table 1**, which indicates that there were no statistically significant differences among the groups in terms of baseline characteristics (P > 0.05).

**Table 1:** Characteristics among the different groups.

Characteristics	Training group N=828	Testing group N=277	Validation group N=276	P value
Blastcyst derived from day3 cells				
8 cells	272 (32.85%)	81 (29.24%)	86 (31.16%)	0.520
non 8 cells	556 (67.15%)	196 (70.76%)	190 (68.84%)	
D3 fragmentation^a^				
≤10%	427 (51.57%)	129 (46.57%)	139 (50.36%)	0.706
11%-25%	381 (46.01%)	141 (50.90%)	131 (47.46%)	
26%-50%	20 (2.42%)	7 (2.53%)	6 (2.17%)	
Blastcyst stage				
3	121 (14.61%)	36 (13.00%)	36 (13.04%)	0.835
4	436 (52.66%)	152 (54.87%)	151 (54.71%)	
5	224 (27.05%)	75 (27.08%)	79 (28.62%)	
6	47 (5.68%)	14 (5.05%)	10 (3.62%)	
ICM				
A	226 (27.29%)	66 (23.83%)	73 (26.45%)	0.785
B	402 (48.55%)	146 (52.71%)	138 (50.00%)	
C	200 (24.15%)	65 (23.47%)	65 (23.55%)	
TE				
A	263 (31.76%)	88 (31.77%)	86 (31.16%)	0.977
B	417 (50.36%)	138 (49.82%)	144 (52.17%)	
C	148 (17.87%)	51 (18.41%)	46 (16.67%)	
Fertilization				
IVF	622 (75.12%)	222 (80.14%)	211 (76.45%)	0.881
ICSI	206 (24.88%)	55 (19.86%)	65 (23.55%)	
Infertility type				
PI	321 (38.77%)	92 (33.21%)	99 (35.87%)	0.228
SI	507 (61.23%)	185 (66.79%)	177 (64.13%)	
First-time transfer				
yes	313 (37.80%)	109 (39.35%)	107 (38.77%)	0.886
no	515 (62.20%)	168 (60.65%)	169 (61.23%)	
Previous parity				
0	602 (72.71%)	195 (70.40%)	205 (74.28%)	0.767
1	205 (24.76%)	75 (27.08%)	68 (24.64%)	
2	20 (2.42%)	7 (2.53%)	3 (1.09%)	
≥3	1 (0.12%)	0 (0.00%)	0 (0.00%)	
Previous abortus				
0	491 (59.30%)	148 (53.43%)	151 (54.71%)	
1	248 (29.95%)	86 (31.05%)	80 (28.99%)	
2	61 (7.37%)	35 (12.64%)	36 (13.04%)	
≥3	28 (3.38%)	8 (2.89%)	9 (3.26%)	
Infertility cause Tubal				
No	183 (22.10%)	71 (25.63%)	69 (25.00%)	0.378
Yes	645 (77.90%)	206 (74.37%)	207 (75.00%)	
Endometriosis				
No	774 (93.48%)	264 (95.31%)	258 (93.48%)	0.527
Yes	54 (6.52%)	13 (4.69%)	18 (6.52%)	
Ovarian causes				
No	627 (75.72%)	194 (70.04%)	208 (75.36%)	0.160
Yes	201 (24.28%)	83 (29.96%)	68 (24.64%)	
Uterine causes				
No	796 (96.14%)	264 (95.31%)	266 (96.38%)	0.783
Yes	32 (3.86%)	13 (4.69%)	10 (3.62%)	
Unexplained				
No	768 (92.75%)	251 (90.61%)	248 (89.86%)	0.237
Yes	60 (7.25%)	26 (9.39%)	28 (10.14%)	
Male factors				
No	583 (70.41%)	198 (71.48%)	175 (63.41%)	0.061
Yes	245 (29.59%)	79 (28.52%)	101 (36.59%)	
Endometrial preparation				
mNC/MS	491 (59.30%)	169 (61.01%)	165 (59.78%)	0.234
HRT/GnRHa-HRT	337 (40.70%)	108 (38.99%)	111 (40.22%)	
Male age at OPU (years)	33.55 ± 5.43	33.89 ± 5.66	33.49 ± 5.46	0.400
Maternal age at OPU (years)	31.74 ± 4.83	32.12 ± 4.94	31.83 ± 4.64	0.554
Intervals between blastocyst thawing and vitrification (years)	1.42 ± 1.57	1.39 ± 1.56	1.50 ± 1.68	0.714
Maternal BMI (kg/m^2^)	21.76 ± 2.78	21.63 ± 2.96	21.94 ± 2.89	0.196
Maternal bFSH (mIU/ml)	6.51 ± 1.62	6.35 ± 1.63	6.33 ± 1.66	0.987
Maternal bLH (mIU/ml)	5.94 ± 3.12	5.97 ± 3.03	5.89 ± 2.79	0.966
Maternal infertility duration (years)	4.48 ± 3.18	4.88 ± 3.52	4.44 ± 3.25	0.598
E_2_ on HCG trigger day (pg/ml)	4717.11 ± 2442.52	4551.14 ± 2082.55	4738.74 ± 2349.22	0.218
Total Gn dose (IU)	2303.21 ± 889.22	2340.26 ± 938.56	2295.74 ± 947.34	0.432
Oocyte retrieval	19.86 ± 7.56	19.80 ± 7.60	19.92 ± 7.56	0.549
Endometrium thickness (mm)	9.65 ± 1.71	9.60 ± 1.65	9.61 ± 1.70	0.767

aD3 fragmentation represents blastocysts derived from embryos with fragmentation observed on day 3 after fertilization.

### 3.2 Logistic regression analysis

**Figure 1:**
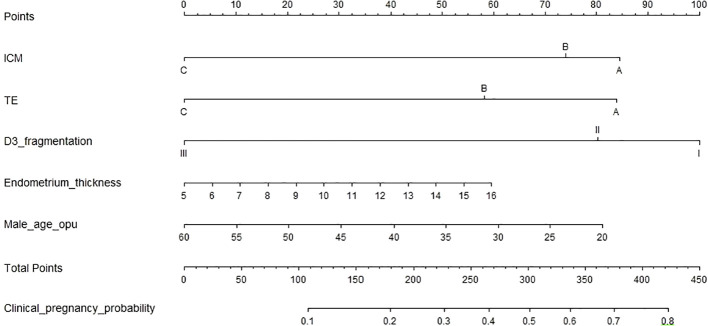
This nomogra predicts the probability of clinical pregnancy in patients undergoing frozen-thaved cycles with single Day 6 blastocyst transfers. To use nomogram, follow these steps: Draw a perpendicular line from the axis of each risk factor up to the line labeled “Points”. Sum the points for all risk factos to obtain the total score. Then, draw a descending line from the “Total Points” axis until it intersects with the lower line, which determines the probability of clinical pregnancy. The optimal thres hold point was determined using a receiver operating characteristic (ROC) curve.

**Table 2** shows the univariate logistic regression results for clinical pregnancy outcomes in single Day 6 blastocyst transfer cycles, identifying ten significant variables (P < 0.05): Day 3 (D3) fragmentation, blastocyst stage, inner cell mass (ICM) grade, trophectoderm (TE) grade, ovarian causes, male and maternal age at oocyte pickup (OPU), total gonadotropin (Gn) dose, oocyte retrieval number, and endometrium thickness. LASSO regression highlighted D3 fragmentation, ICM grade, TE grade, male age at OPU, and endometrium thickness as key independent predictors. In the multivariate logistic regression model (**Table 3**), the independent predictors were: D3 fragmentation (11%–25% vs. <10%, OR 0.80, 95% CI: 0.60–1.07, P = 0.132; 25%–50% vs. <10%, OR 1.06, 95% CI: 0.98–1.15, P = 0.142), ICM grade (Grade B vs. A, OR 0.89, 95% CI: 0.61–1.29, P = 0.535; Grade C vs. A, OR 0.38, 95% CI: 0.23–0.63, P < 0.001), TE grade (Grade B vs. A, OR 0.75, 95% CI: 0.52–1.08, P = 0.119; Grade C vs. A, OR 0.39, 95% CI: 0.23–0.64, P < 0.001), male age at OPU (OR 0.98, 95% CI: 0.95–1.00, P = 0.095), and endometrium thickness (OR 1.06, 95% CI: 0.98–1.15, P = 0.142). A nomogram (**Figure 1**) was developed using these five predictors to calculate clinical pregnancy probabilities, offering a practical tool for individualized patient care.

**Table 2:** Univariate analysis in the training group.

Variables	Odds Ratio	95% Confidence Interval	P value
Blastcyst derived from day3 cells			
8 cells			
non 8 cells	0.85	(0.63-1.14)	0.27
D3 fragmentation			
≤10%			
11%-25%	0.69	(0.52-0.92)	0.01^*^
26%-50%	0.74	(0.29-1.84)	0.51
Blastcyst stage			
3			
4	1.8	(1.17-2.77)	0.007^*^
5	2.11	(1.32-3.37)	0.002^*^
6	1.68	(0.84-3.37)	0.14
ICM			
A			
B	0.71	(0.51-0.99)	0.042^*^
C	0.31	(0.20-0.46)	<0.001^**^
TE			
A			
B	0.65	(0.48-0.89)	0.007^*^
C	0.33	(0.21-0.50)	<0.001^**^
Fertilization			
IVF			
ICSI	1	(0.73-1.37)	0.99
Infertility type			
PI			
SI	0.78	(0.59-1.03)	0.08
First-time transfer			
yes			
no	1.1	(0.82-1.45)	0.53
Previous parity			
0			
1	1.14	(0.82-1.57)	0.44
2	0.44	(0.16-1.24)	0.12
≥3	1.33	(0.27-6.66)	0.73
Previous abortus			
0			
1	0.73	(0.54-1.00)	0.052
2	0.86	(0.50-1.48)	0.59
≥3	0.55	(0.24-1.24)	0.15
Infertility cause Tubal			
No			
Yes	0.8	(0.58-1.11)	0.18
Endometriosis			
No			
Yes	0.9	(0.51-1.57)	0.70
Ovarian causes			
No			
Yes	0.68	(0.49-0.95)	0.023^*^
Uterine causes			
No			
Yes	1.51	(0.74-3.07)	0.25
Unexplained			
No			
Yes	1	(0.59-1.71)	0.99
Male factors			
No			
Yes	1.18	(0.87-1.59)	0.28
Endometrial preparation			
mNC/MS			
HRT/GnRHa-HRT	1.28	(0.97-1.70)	0.08
Male age at OPU	0.96	(0.93-0.98)	0.002^*^
Maternal age at OPU	0.96	(0.93-0.99)	0.008^*^
Intervals between blastocyst thawing and vitrification	1	(1.00-1.00)	0.37
Maternal BMI	0.99	(0.94-1.04)	0.63
Maternal bFSH	0.98	(0.90-1.07)	0.70
Maternal bLH	1.03	(0.99-1.08)	0.20
Maternal infertility duration	0.98	(0.94-1.03)	0.49
E_2_ on HCG trigger day	1	(1.00-1.00)	0.29
Total Gn dose	1	(1.00-1.00)	0.042^*^
Oocyte retrieval	1.02	(1.00-1.04)	0.048^*^
Endometrium thickness	1.13	(1.05-1.23)	0.002^*^

'*' represents a p-value less than 0.05.

**Table 3:** Multivariate logistic regression model in the training group.

Variables	Odds Ratio	95% Confidence Interval	P value
D3 fragmentation			
≤10%			
11%-25%	0.8	(0.60-1.07)	0.132
26%-50%	0.32	(0.10-1.01)	0.052
ICM			
A			
B	0.89	(0.61-1.29)	0.535
C	0.38	(0.23-0.63)	<0.001^**^
TE			
A			
B	0.75	(0.52-1.08)	0.119
C	0.39	(0.23-0.64)	<0.001^**^
Male age at OPU	0.98	(0.95-1.00)	0.095
Endometrium thickness	1.06	(0.98-1.15)	0.142

'**' represents a p-value less than 0.001.

### 3.3 Validation and performance of the clinical pregnancy prediction model

**Figure 2:**
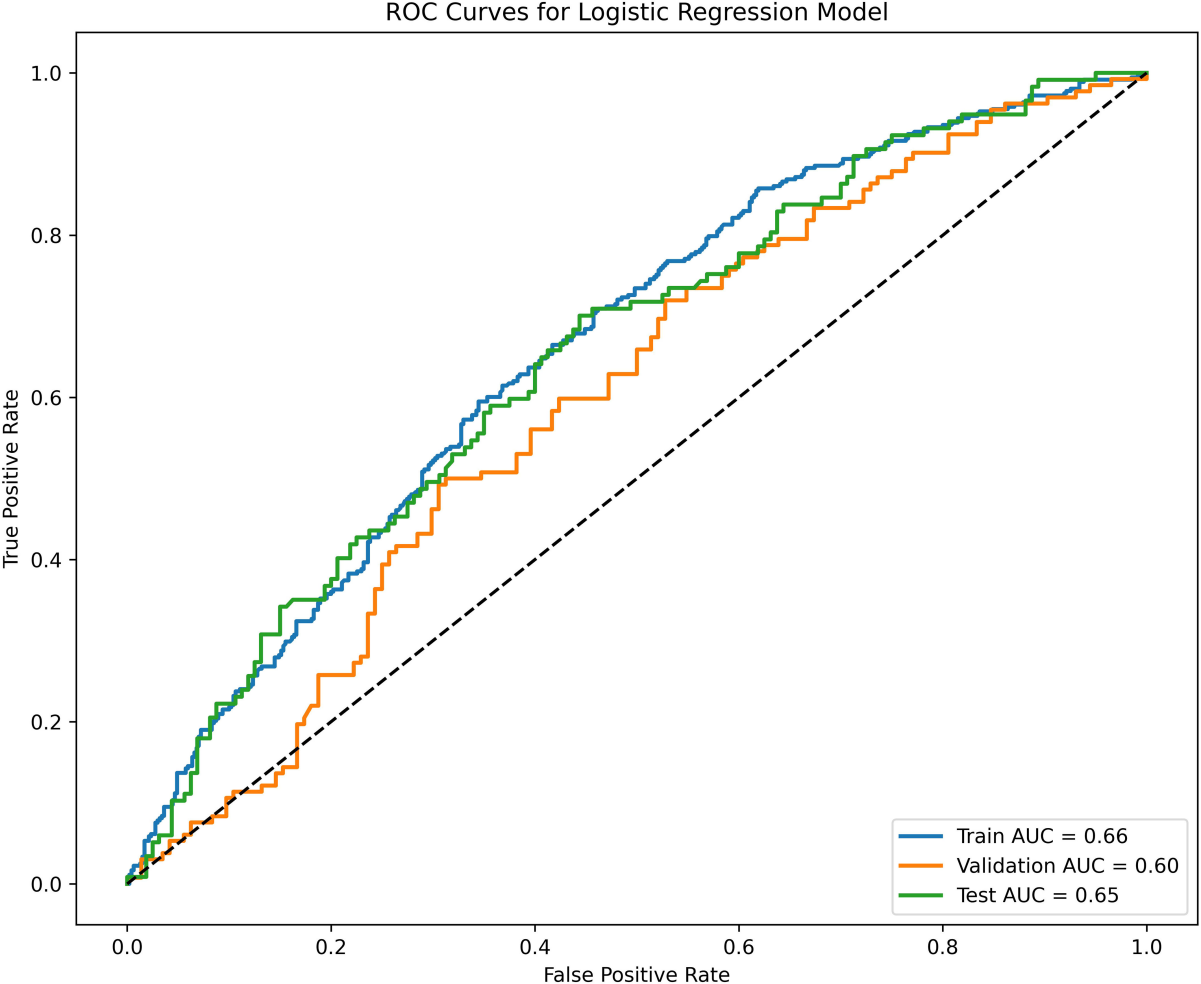
Receiver operating characteristic (ROC) curves and calibration plots for the training, testing, and validation groups. The blue line represents the training group, with an AUC of 0.66. The green line represents the testing group, with an AUC of 0.65. The orange line represents the validation group, with an AUC of 0.60.

**Figure 3:**
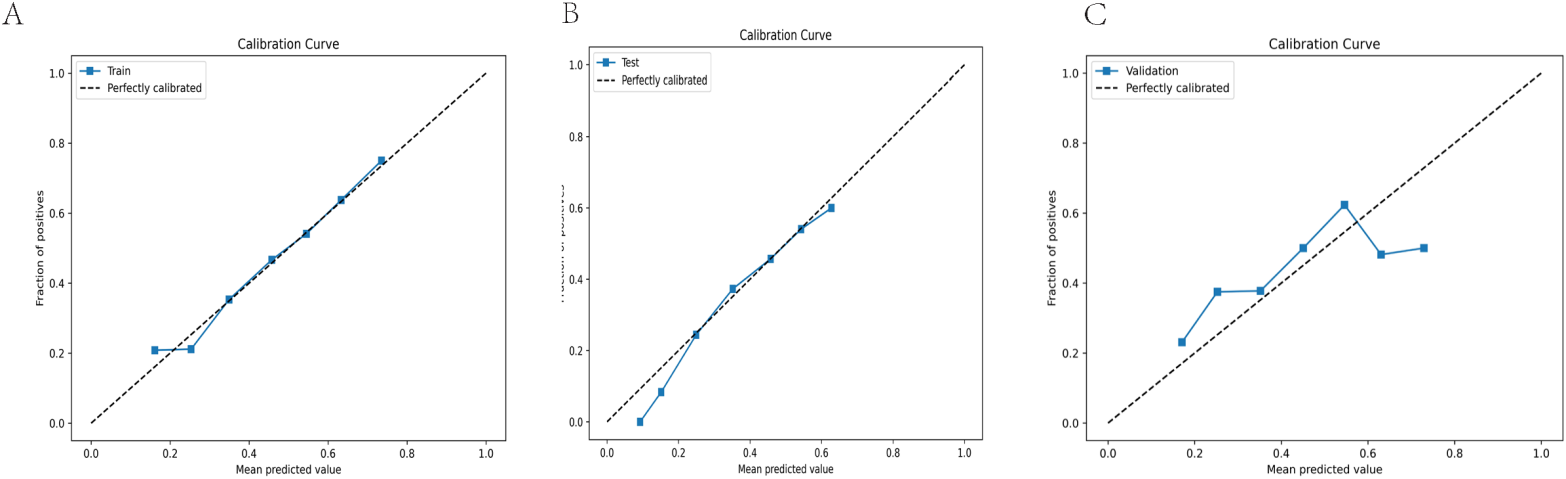
Calibration curves are employed to assess the accuracy of the model’s calibration. The horizontal axis represents the predicted probability produced by the model, while the vertical axis shows the observed probability of clinical pregnancy. An ideal line with a 45° slope symbolizes perfect prediction, where the predicted probability matches the observed probability precisely. A slope closer to 1.00 indicates more accurate calibration power of the prediction model. **(A)** Calibration curve for the training group (Slope = 0.988) **(B)** Calibration curve for the testing group (Slope = 0.871). **(C)** Calibration curve for the validation group (Slope = 1.263).

**Figure 4:**
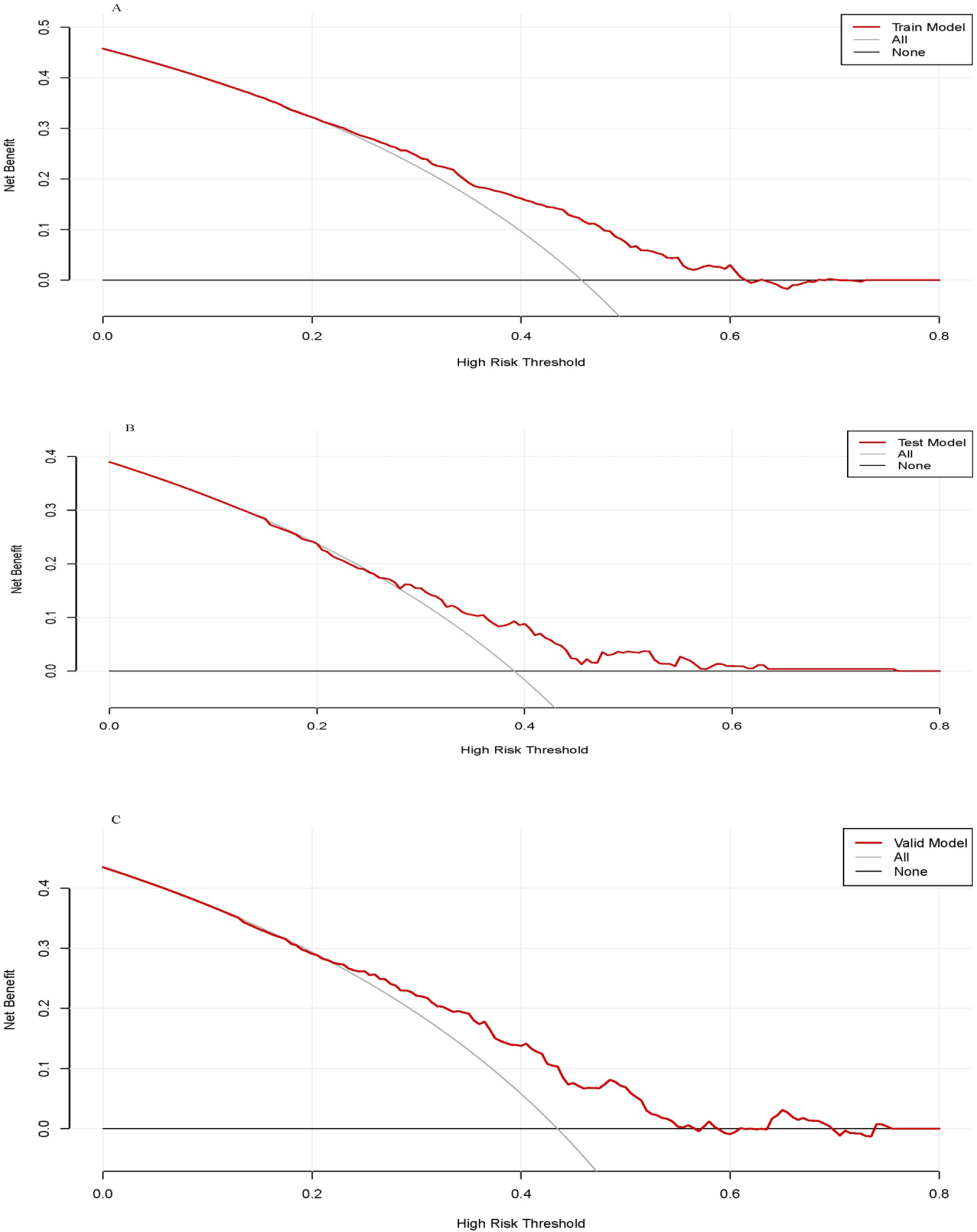
The decision curve analysis of the training group **(A)**, testing group **(B)** and validation group **(C)**.

The performance of the clinical pregnancy prediction model was evaluated using the Area Under the Curve (AUC), calibration plots, and decision curve analysis (DCA). The AUC values were 0.66, 0.65, and 0.60 for the training, testing, and validation groups, respectively, indicating acceptable predictive capability (**Figure 2**). Calibration plots showed slopes of 0.988, 0.871, and 1.263 for the three groups (**Figures 3A–C**), demonstrating good alignment between predicted and observed outcomes. DCA further confirmed the model’s clinical utility, showing substantial net benefit across the training, testing, and validation groups, as reflected in favorable positions on the decision curves (**Figures 4A–C**).

## 4 Discussion

### 4.1 Multivariate analysis affecting clinical pregnancy in single Day 6 blastocyst transfers

The objective of this study was to create a predictive model to estimate clinical pregnancy likelihood for patients undergoing single Day 6 frozen-thawed blastocyst transfers. We conducted a two-center retrospective cohort analysis of 1,381 transfer cycles from June 2016 to December 2022, aiming to identify significant predictors influencing clinical pregnancy outcomes. The use of data from two reproductive centers improves the model’s representation of diverse clinical practices, thus enhancing its generalizability across wider populations. Nonetheless, individual patient variability and slight protocol differences between centers continue to present challenges, which are addressed in the Limitations section.

Our analysis identified several independent predictors, including inner cell mass (ICM) grade, trophectoderm (TE) grade, Day 3 (D3) fragmentation, endometrial thickness, and male age at oocyte pickup (OPU), aligning with existing research findings. These results underscore the importance of embryo quality in achieving successful clinical outcomes (21, 22). Both ICM and TE grading serve as indicators of key embryo morphological characteristics, with higher grades linked to improved implantation potential and clinical pregnancy rates. In our study, embryos with Grade C ICM and TE scores were associated with lower pregnancy success, consistent with prior research that suggests lower-grade embryos have limited developmental capacity (23).This highlights the critical role of precise morphological evaluation in optimizing ART outcomes.

Day 3 fragmentation also emerged as a crucial predictor, as it is widely acknowledged as a measure of embryo quality. Increased fragmentation indicates abnormal cell division, leading to reduced implantation potential and impaired embryonic development (24). Recent research further suggests that extracellular vesicles (EVs) may modulate the embryonic microenvironment and potentially influence embryo fragmentation through intercellular signaling pathways (25). Our findings show that embryos with fragmentation levels below 10% demonstrated better clinical outcomes compared to those with higher fragmentation (26).Thus, Day 3 fragmentation serves as a valuable metric in embryo selection, assisting embryologists in identifying embryos with greater developmental potential.

Endometrial thickness also proved to be a significant predictor. While a thicker endometrium generally enhances implantation success (27), excessive thickness can negatively impact implantation, often due to underlying conditions such as polyps or fibroids. Identifying an optimal range for endometrial thickness is therefore critical for enhancing pregnancy outcomes in fertility treatments.

Lastly, male age at OPU was identified as a predictor, underscoring the increasing acknowledgment of paternal factors in fertility outcomes. Advanced paternal age is linked to decreased sperm quality and heightened miscarriage risk (28). These findings emphasize the importance of a holistic fertility assessment that considers both partners, which can lead to more effective treatment strategies and improved clinical outcomes.

### 4.2 Development and validation of the nomogram prediction model

Our study successfully developed a well-calibrated nomogram to predict clinical pregnancy probabilities following Day 6 frozen-thawed blastocyst transfers, with AUC values of 0.66, 0.65, and 0.60 for training, testing, and validation groups, respectively. These values indicate a satisfactory level of predictive accuracy, consistent with prior ART predictive model studies, where AUCs generally range from 0.59 to 0.8 (29). The clinical value of predictive models is increasingly underscored, especially through decision curve analysis (DCA), which evaluates a model’s capacity to improve clinical decision-making by benefiting patients (30).Our model’s validity was further confirmed with calibration curves, which demonstrated strong concordance between predicted and observed clinical pregnancy probabilities. Calibration is a vital component in prediction model evaluation, measuring how accurately a model estimates absolute risks (31). A 2022 article in JAMA emphasized the importance of including calibration curves in clinical studies to ensure model applicability in real-world settings.

In our study, participants were randomly divided into training, testing, and validation groups in a 6:2:2 ratio, demonstrating consistent model performance across all phases. This consistency underscores the model’s reliability for clinical use. Decision curve analysis (DCA) affirmed the model’s substantial net benefit over various threshold probabilities, supporting its practical value for clinicians. Given that lifestyle factors significantly impact reproductive outcomes in cases such as PCOS, combining personalized lifestyle interventions with predictive models may further optimize ART results (32). This approach is in line with evidence showing that lifestyle interventions improve both metabolic and reproductive outcomes in ART settings. By enhancing clinical decision-making, the model fosters personalized treatment plans and optimizes resource allocation in clinical practice.

### 4.3 Limitations and future directions

Firstly, the retrospective design of this study might introduce potential selection bias due to individual patient variability and differences in treatment protocols across the two participating centers. Variations in clinical practices may affect outcomes, limiting the generalizability of the model to other settings. Future prospective studies should adopt standardized protocols across centers and collect real-time data to minimize bias and improve the reliability of predictions. Secondly, the model does not account for genetic factors or external influences such as psychological stress, lifestyle, and environmental factors, which can impact embryo quality, implantation, and pregnancy success. These unmeasured factors may play a role in the variability of outcomes, even when patients share similar independent predictors. Future research should integrate genetic data and assess external factors to enhance the model’s predictive power and provide a more comprehensive understanding of reproductive outcomes. Thirdly, although the model demonstrated consistent performance across the training, testing, and validation groups, external validation in diverse populations and clinical settings is essential to confirm its generalizability. The model’s application in multi-center prospective studies will help identify additional influencing factors, refine the model, and ensure its relevance across varying healthcare environments. Such efforts will enhance the model’s utility in supporting personalized fertility care and guiding clinical decisions more effectively.

## 5 Conclusion

This two-center retrospective cohort study developed a well-calibrated predictive model that accurately estimates the probability of clinical pregnancy, offering significant clinical implications for fertility treatments. The model serves as a robust tool for predicting clinical outcomes in frozen-thawed single Day 6 blastocyst transfer cycles. By integrating key embryological and clinical parameters—including inner cell mass (ICM) grade, trophectoderm (TE) grade, Day 3 (D3) fragmentation, and male age at oocyte pick-up (OPU)—the model enhances both patient counseling and clinical decision-making. This comprehensive approach improves the precision of predictions, helping clinicians make more informed decisions, and ultimately enhances the efficiency and success of assisted reproductive technologies (ART).

## Data Availability

The raw data supporting the conclusions of this article will be made available by the authors, without undue reservation.

## References

[1] Raja E-A Bhattacharya S Maheshwari A McLernon DJA. Comparison of perinatal outcomes following fresh blastocyst or cleavage stage embryo transfer in singletons and twins and between singleton siblings. Hum Reprod Open. (2023) 2023:hoad003. doi: 10.1093/hropen/hoad003 36909797 PMC9995092

[2] Voelker R. Researchers in Canada call for policy to mandate single-embryo transfer in IVF. JAMA. (2011) 305:1848. doi: 10.1001/jama.2011.602 21558511

[3] Finelli R Terribile M Wilding M Nargund G. P-239 comparison of reproductive outcomes for cleavage- and blastocyst-stage frozen embryo transfer: A retrospective study. Hum Reprod. (2023) 38:dead093.597. doi: 10.1093/humrep/dead093.597

[4] Wilson M Hartke K Kiehl M Rodgers J Brabec C Lyles R. Integration of blastocyst transfer for all patients. Fertil Steril. (2002) 77:693–6. doi: 10.1016/S0015-0282(01)03235-6 11937117

[5] Kulkarni AD Jamieson DJ Jones HW Jr Kissin DM Gallo MF Macaluso M. Fertility treatments and multiple births in the United States. N Engl J Med. 369(23):2218–25. doi: 10.1056/NEJMoa1301467 24304051

[6] Abdala A Elkhatib I Bayram A El-Damen A Melado L Nogueira D. Reproductive outcomes with delayed blastocyst development: the clinical value of day 7 euploid blastocysts in frozen embryo transfer cycles. Zygote. (2023) 31:588–95. doi: 10.1017/S0967199423000485 37955175

[7] Park DS Kim JW Chang EM Lee WS Yoon TK Lyu SW. Obstetric, neonatal, and clinical outcomes of day 6 vs. Day 5 vitrified-warmed blastocyst transfers: retrospective cohort study with propensity score matching. Front Endocrinol. (2020) 11:499. doi: 10.3389/fendo.2020.00499 PMC7418454 32849288

[8] Bourdon M Pocate-Cheriet K Finet De Bantel A Grzegorczyk-Martin V Amar Hoffet A Arbo E. Day 5 versus day 6 blastocyst transfers: A systematic review and meta-analysis of clinical outcomes. Hum Reprod. (2019) 34:1948–64. doi: 10.1093/humrep/dez163 PMC7967799 31644803

[9] Cai H Mol B Gordts S Wang H Shi J. Elective single versus double blastocyst-stage embryo transfer in women aged 36 years or older: A retrospective cohort study. Hum Fertil. (2023) 26:1185–94. doi: 10.1080/14647273.2022.2153348 36719262

[10] Wu T-F Chen M-J Lee M-S Huang C-C Ho S-T Cheng E-H. Comparison of clinical outcome between day 5 and day 6 single blastocyst transfers in cycles undergoing preimplantation genetic testing for aneuploidy. Taiwan J Obstet Gynecol. (2023) 62:429–33. doi: 10.1016/j.tjog.2023.03.005 37188448

[11] Hu J Zheng J Li J Shi H Wang H Zheng B. D6 high-quality expanded blastocysts and D5 expanded blastocysts have similar pregnancy and perinatal outcomes following single frozen blastocyst transfer. Front Endocrinol. (2023) 14:1216910. doi: 10.3389/fendo.2023.1216910 PMC10666767 38027138

[12] Melado Vidales L Lawrenz B Vitorino RL Patel R Ruiz FJ Marques LM. Clinical and laboratory parameters associated with cycle outcomes in patients undergoing euploid frozen blastocyst transfer. Reprod Biomed Online. (2023) 46:917–25. doi: 10.1016/j.rbmo.2023.02.014 37062636

[13] Crosby D O’Brien Y Glover L Martyn F Wingfield M. Influence of body mass index on the relationship between endometrial thickness and pregnancy outcome in single blastocyst frozen embryo transfer cycles. Hum Fertil. (2020) 23:32–7. doi: 10.1080/14647273.2018.1504324 30221570

[14] Ai J Jin L Zheng Y Yang P Huang B Dong X. The morphology of inner cell mass is the strongest predictor of live birth after a frozen-thawed single embryo transfer. Front Endocrinol. (2021) 12:621221. doi: 10.3389/fendo.2021.621221 PMC7943864 33716973

[15] Ozgur K Berkkanoglu M Bulut H Donmez L Isikli A Coetzee K. Blastocyst age, expansion, trophectoderm morphology, and number cryopreserved are variables predicting clinical implantation in single blastocyst frozen embryo transfers in freeze-only-IVF. J Assist Reprod Genet. (2021) 38:1077–87. doi: 10.1007/s10815-021-02110-7 PMC8190197 33594625

[16] Chen H Wu S Su W Zhao J Wu Y. Comparison of pregnancy outcomes among patients of different ages who underwent frozen-thawed high-quality single blastocyst transfer. BMC Pregnancy Childbirth. (2024) 24:276. doi: 10.1186/s12884-024-06451-w 38622514 PMC11017700

[17] Peng C-YJ Lee KL Ingersoll GM. An introduction to logistic regression analysis and reporting. J Educ Res. (2002) 96:3–14. doi: 10.1080/00220670209598786

[18] Peluso C Oliveira RD Laporta GZ Christofolini DM Fonseca FLA Laganà AS. Are ovarian reserve tests reliable in predicting ovarian response? Results from a prospective, cross-sectional, single-center analysis. Gynecol Endocrinol. (2021) 37:358–66. doi: 10.1080/09513590.2020.1786509 32613875

[19] Gardner DK Schoolcraft WB. Culture and transfer of human blastocysts. Curr Opin Obstet Gynaecol. (1999) 11:307–11. doi: 10.1097/00001703-199906000-00013 10369209

[20] Cozzolino M Vitagliano A Di Giovanni MV Laganà AS Vitale SG Blaganje M. Ultrasound-guided embryo transfer: summary of the evidence and new perspectives. A systematic review and meta-analysis. Reprod Biomed Online. (2018) 36:524–42. doi: 10.1016/j.rbmo.2018.01.015 29576332

[21] Zhao Y-Y Yu Y Zhang X-W. Overall blastocyst quality, trophectoderm grade, and inner cell mass grade predict pregnancy outcome in euploid blastocyst transfer cycles. Chin Med J (Engl.). (2018) 131:1261–7. doi: 10.4103/0366-6999.232808 PMC5987494 29786036

[22] Van Den Abbeel E Balaban B Ziebe S Lundin K Cuesta MJG Klein BM. Association between blastocyst morphology and outcome of single-blastocyst transfer. Reprod Biomed Online. (2013) 27:353–61. doi: 10.1016/j.rbmo.2013.07.006 23953585

[23] Shen X Long H Gao H Guo W Xie Y Chen D. The valuable reference of live birth rate in the single vitrified-warmed BB/BC/CB blastocyst transfer: the cleavage-stage embryo quality and embryo development speed. Front Physiol. (2020) 11:1102. doi: 10.3389/fphys.2020.01102 33013471 PMC7511572

[24] Liu J Zhou Y Tong L Wang X Li Y Wang H. Developmental potential of different embryos on day 3: A retrospective study. J Obstet Gynaecol. (2022) 42:3322–7. doi: 10.1080/01443615.2022.2125291 36149236

[25] Zhou G Gu Y Zhou F Zhang M Zhang G Wu L. The emerging roles and therapeutic potential of extracellular vesicles in infertility. Front Endocrinol. (2021) 12:758206. doi: 10.3389/fendo.2021.758206 PMC8569852 34745016

[26] Chae-Kim J Waggener K Gavrilova-Jordan L. Defragmentation of *in-vitro* fertilization blastocyst stage embryos leading to rescued blastocyst expansion and clinical pregnancy. Clin Obstet Gynecol Reprod Med. (2020) 6(1-3). doi: 10.15761/COGRM.1000317

[27] Xu J Zhang S Jin L Mao Y Shi J Huang R. The effects of endometrial thickness on pregnancy outcomes of fresh IVF/ICSI embryo transfer cycles: an analysis of over 40,000 cycles among five reproductive centers in China. Front Endocrinol. (2022) 12:788706. doi: 10.3389/fendo.2021.788706 PMC8818785 35140680

[28] Kong P Liu Y Zhu Q Yin M Teng X. Effect of male age on pregnancy and neonatal outcomes in the first frozen-thawed embryo transfer cycles of IVF/ICSI treatment. Andrology. (2021) 9:1540–8. doi: 10.1111/andr.13031 33961339

[29] Curchoe CL Flores-Saiffe Farias A Mendizabal-Ruiz G Chavez-Badiola A. Evaluating predictive models in reproductive medicine. Fertil Steril. (2020) 114:921–6. doi: 10.1016/j.fertnstert.2020.09.159 33160514

[30] Van Calster B Wynants L Verbeek JFM Verbakel JY Christodoulou E Vickers AJ. Reporting and interpreting decision curve analysis: A guide for investigators. Eur Urol. (2018) 74:796–804. doi: 10.1016/j.eururo.2018.08.038 30241973 PMC6261531

[31] Alba AC Agoritsas T Walsh M Hanna S Iorio A Devereaux PJ. Discrimination and calibration of clinical prediction models: users’ Guides to the medical literature. JAMA. (2017) 318:1377. doi: 10.1001/jama.2017.12126 29049590

[32] Gu Y Zhou G Zhou F Wu Q Ma C Zhang Y. Life modifications and PCOS: old story but new tales. Front Endocrinol. (2022) 13:808898. doi: 10.3389/fendo.2022.808898 PMC9045543 35498415

